# Complete Genome Sequence of a Classical Swine Fever Virus Isolate Belonging to a New Subgenotype, 2.1c, from Guangxi Province, China

**DOI:** 10.1128/genomeA.00311-15

**Published:** 2015-06-04

**Authors:** Weixing Shao, Yaohua Huang, Shuang Liu, Faxing Wu, Zhi Zhang, Yaqin Dong, Xiaocheng Li

**Affiliations:** China Animal Health and Epidemiology Center, Division of Surveillance of Livestock Diseases, Qingdao, China

## Abstract

The complete genome sequence of a field isolate of classical swine fever strain (CSFV), GXF29/2013, was determined in this study. This strain was originally isolated from infected pigs in Guangxi Province, China. The most significant difference in the amino acid sequence of the polyprotein from subgenotypes 2.1a and 2.1b is an SPA→APV amino acid substitution at positions 88 and 90 in the E2 protein.

## GENOME ANNOUNCEMENT

Classical swine fever (CSF), which causes heavy losses in the swine industry, is one of the Office International des Epizooties (OIE) notifiable diseases caused by classical swine fever virus (CSFV) (1). CSFV is a positive-sense RNA virus belonging to the genus *Pestivirus* within the family *Flaviviridae*. The CSFV genome is approximately 12.3 kb in length and encodes a single polyprotein that is co- and posttranslationally cleaved to form mature structural and nonstructural proteins (2, 3). Phylogenetic analysis based on sequence data sets of the 5′ untranslated region (UTR), E2 envelope glycoprotein gene, and nonstructural 5B (NS5B) polymerase gene divides CSFV into three genotypes (I, II, and III), with each being further divided into three or four subgenotypes (1.1 to 1.3, 2.1 to 2.3, and 3.1 to 3.4) (4). CSFV subgenotype 2.1 has been further divided into 2.1a and 2.1b (5, 6). In mainland China, CSFVs of subgenotype 2.1b have predominated since the 1990s (7–9).

Here, we describe the complete genome sequence of a field isolate of CSFV, GXF29/2013, which was isolated from an intensive pig farm in Guangxi Province, China, in 2013. The full nucleotide sequence was obtained from four overlapping fragments amplified by reverse transcription-PCR. Four amplified PCR products were purified and respectively cloned into vector PMD-19, and the recombinant plasmids were sequenced with an ABI 3730 XL genome sequencer (Sangon Company, Shanghai, China). The sequences were assembled and manually edited to produce the final genome sequence.

The complete genome sequence of the isolate GXF29/2013 is 12,296 nucleotides (nt) in length, with a 5′ UTR of 373 nt and a 3′ UTR of 226 nt. A single large open reading frame (ORF) is 11,679 nt between nucleotide positions 374 and 12070 and is capable of coding for a polyprotein of 3,898 amino acids.

Comparing the fragment of E2 of GXF29/2013 with 28 reference strains, except CSF0705 (Cuba), CSF1057 (Cuba), and GXGL-1/06 (10), phylogenetic analysis located GXF29/2013 in a new CSFV subgroup, 2.1c. The difference in the sequences of the fragment of E2 between GXF29/2013 with those of subgroup 2.1c CSFVs was 2.2 to ~3.3%. However, a more significant level of nucleotide difference was found, at 9.1 to ~11.6% and 9.7 to ~13.7% with those of subgroup 2.1a and 2.1b CSFVs, respectively, indicating that GXF29/2013 is more closely related to subgroup 2.1a than to 2.1b. The most significant difference in the polyproteins of GXF29/2013 from another subgroup is the SPA→APV amino acid substitution at positions 88 and 90 in the E2 protein. There is 1 amino acid substitution at position 343 from V to I in the subgroup 2.1c CSFVs, including GXF29/2013, which can be a marker of subgroup 2.1c CSFVs. These findings provide strong evidence for substantial genetic variation within subgroup 2.1 CSFVs, which may have implications for the prevention and control of CSFV infection in pigs. The complete genome sequence of CSFV strain GXF29/2013 should allow for further studies on genetic diversity and the relationship of genotype 2.1 strains.

### Nucleotide sequence accession number. 

The complete genome sequence of CSFV GXF29/2013 has been deposited in GenBank under the accession no. KP233070.
